# Application of Deferoxamine in Tissue Regeneration Attributed to Promoted Angiogenesis

**DOI:** 10.3390/molecules29092050

**Published:** 2024-04-29

**Authors:** Haijun Shen, Yane Ma, Yi Qiao, Chun Zhang, Jialing Chen, Ran Zhang

**Affiliations:** 1 Department of Preventive Medicine and Public Health Laboratory Science, School of Medicine, Jiangsu University, 301 Xuefu Road, Zhenjiang 212013, China; mye2515@163.com (Y.M.); 15956107312@163.com (Y.Q.); zzzzc1008@163.com (C.Z.); cjl991117@163.com (J.C.); 2 Jiangsu Cancer Hospital, Jiangsu Institute of Cancer Research, The Affiliated Cancer Hospital of Nanjing Medical University, No. 42 Baiziting, Nanjing 210009, China

**Keywords:** deferoxamine, angiogenesis, wound healing, diabetes ulcer, bone repair

## Abstract

Deferoxamine, an iron chelator used to treat diseases caused by excess iron, has had a Food and Drug Administration-approved status for many years. A large number of studies have confirmed that deferoxamine can reduce inflammatory response and promote angiogenesis. Blood vessels play a crucial role in sustaining vital life by facilitating the delivery of immune cells, oxygen, and nutrients, as well as eliminating waste products generated during cellular metabolism. Dysfunction in blood vessels may contribute significantly to the development of life-threatening diseases. Anti-angiogenesis therapy and pro-angiogenesis/angiogenesis strategies have been frequently recommended for various diseases. Herein, we describe the mechanism by which deferoxamine promotes angiogenesis and summarize its application in chronic wounds, bone repair, and diseases of the respiratory system. Furthermore, we discuss the drug delivery system of deferoxamine for treating various diseases, providing constructive ideas and inspiration for the development of new treatment strategies.

## 1. Introduction

Iron is an essential trace element in the human body and plays an important role in biological activities such as oxygen transport, oxygen sensing, electron sensing, electron transfer, energy metabolism, and DNA synthesis [1]. However, excessive iron can lead to diseases such as hemochromatosis, thalassemia, myelodysplastic syndrome, aplastic anemia, etc. The iron-chelating drug deferoxamine (DFO) is commonly used in the treatment of such diseases [2]. DFO that has been approved for use by the Food and Drug Administration (FDA) is a natural product extracted from the fermentation liquor of *Streptococcus* spp. [3].

**Figure 1:**
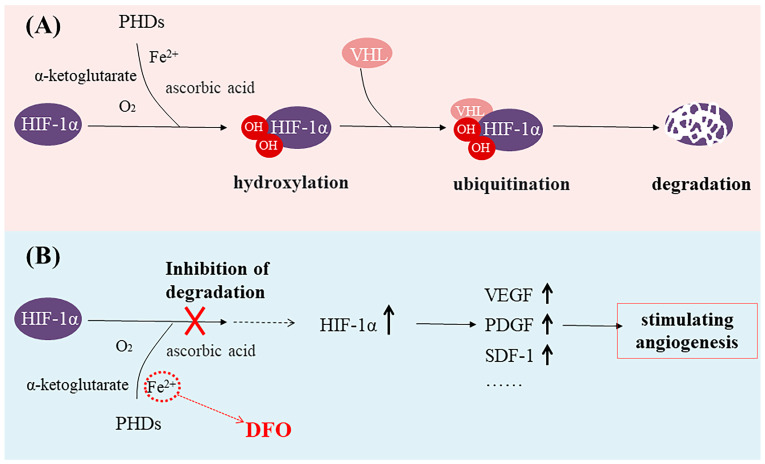
(**A**) The prolyl hydroxylase domain (PHD) utilizes O_2_ and α-ketoglutarate as substrates to hydroxylate two proline residues of hypoxia-inducible factor-1α (HIF-1α), leading to the degradation of HIF-1α. (**B**) Deferoxamine (DFO) binds to Fe^2+^, makes PHD enzymes inactive, and stabilizes the expression of HIF-1α.

Iron is closely associated with inflammation [4,5,6]. During inflammation, the degradation of ferroportin increases, resulting in reduced iron excretion and elevated intracellular iron concentrations and ultimately leading to iron toxicity in cells and tissues [6,7,8]. DFO can bind to unliganded or incompletely liganded iron, rendering the ion inert and preventing its reaction with peroxides which, in turn, mitigates oxidative damage to tissues and alleviates oxidative stress [9,10]. Thus, DFO exhibits potent anti-inflammatory effects as an iron chelator and represents a promising therapeutic approach for mitigating inflammation in various autoimmune and inflammatory disorders [11]. In addition, studies have shown that Fe(II) in the prolyl hydroxylase domain (PHD) catalytic center can be exchanged or chelated by three hydroxamic acid groups of DFO, making PHD enzymes inactive [12]. Because PHD is a hypoxia-inducible factor (HIF) prolyl hydroxylase, it is known to play an important role in oxygen regulation in the physiological network. Hypoxia-inducible factor-1α (HIF-1α) is an oxygen-sensitive molecule [13,14,15,16]. The expression of HIF-1α is upregulated in hypoxic conditions and subsequently regulates multiple target genes [17,18,19,20,21]. The PHD utilizes O_2_ and α-ketoglutarate as substrates to hydroxylate two proline residues of HIF-1α [22,23,24]. Then, the Von Hippel–Lindau protein (VHL) swiftly degrades the hydroxylated HIF-1α [25,26] (Figure 1A). Thus, HIF-1α-mediated gene transcription is inhibited [22]. DFO is able to activate and stabilize a hypoxic HIF-1α pathway by rendering PHD inactive [27,28]. Then, upregulated HIF-1α expression can increase the expression of vascular endothelial growth factor (VEGF, a key signaling molecule in the induction of angiogenesis), platelet-derived growth factor (PDGF), stromal cell-derived factor-1 (SDF-1), and other growth factors, thus stimulating angiogenesis [29,30,31,32,33] (Figure 1B). Numerous studies have demonstrated that DFO, functioning as an iron chelator, can effectively induce the accumulation of HIF-1α, subsequently leading to a significant promotion in endothelial tube formation, cell proliferation, and migration [34,35,36].

The blood vasculature is a closed circulatory system and includes networks of arteries, veins, and capillaries [37]. They play a crucial role in sustaining vital life by facilitating the delivery of immune cells, oxygen, and nutrients, as well as eliminating waste products generated during cellular metabolism [38,39,40,41,42]. The endothelial cells (ECs) are enveloped by mural cells to varying degrees to form blood vessels in various circulatory network locations. Endothelial cells (ECs) line the innermost layer of all of these vessels and exhibit a high degree of heterogeneity among different sections of the vasculature. They play an important role in sensing the circulating environment and responding to extrinsic signals [38]. The process of blood vessel development is intricate, with our current understanding indicating that endothelial cells are the earliest differentiated blood vessel cells during embryonic development and play a pivotal role in the formation of blood vessel walls and the establishment of complete blood vessel networks [43,44,45,46]. In adult organisms, ECs rarely proliferate and remain dormant, but they retain the ability to rapidly form new blood vessels in nutrient-deficient, ischemic/hypoxic environments to restore blood flow (providing oxygen and nutrients) in order to support tissue growth and function [47]. VEGF is implicated in multiple steps of vascular EC development [48] and is a key signaling molecule in the induction of angiogenesis. DFO is able to render the PHD inactive to activate and stabilize HIF-1α in order to increase the expression of VEGF in cells (such as stem cells, human dermal fibroblast cells, and human umbilical vein endothelial cells) [27,28]. VEGF can trigger quiescent ECs to become activated [47] in order to promote cell proliferation and migration and thus promote angiogenesis. 

Dysfunction in blood vessels may significantly contribute to the development of life-threatening diseases [49]. Anti-angiogenesis therapy and pro-angiogenesis/angiogenesis strategies have been frequently recommended for various diseases [47]. Chronic, non-healing wounds are a persistent medical problem, and reduced blood vessel growth is a key reason many chronic wounds are difficult to heal [50,51,52]. Thus, targeted angiogenesis therapy is playing an increasingly important role as a therapeutic strategy for wound healing [50,53,54]. In the skeletal system, the local vascular system is actively involved in bone formation and bone resorption [55,56]. Angiogenesis plays a central role in bone reconstruction by providing oxygen, minerals, nutrients, and growth factors to the injured microenvironment [57,58]. Angiogenesis also plays a pivotal role in the intricate process of fetal lung development and subsequent tissue regeneration following lung transplantation. Based on the role of DFO in promoting angiogenesis, this review discusses the application of DFO in various diseases and provides constructive ideas and enlightenment for the development of more therapeutic strategies for DFO.

## 2. Chronic Wounds

**Figure 2:**
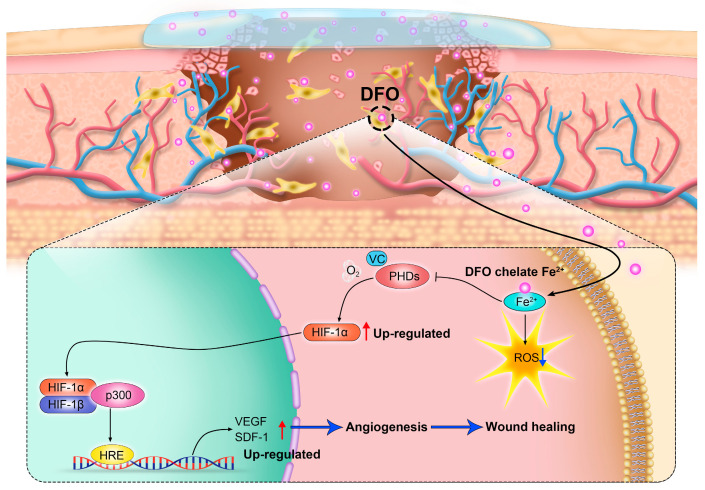
By regulating the hypoxia-inducible factor-1α (HIF-1α) signaling pathway, deferoxamine (DFO) promotes angiogenesis and accelerates wound healing.

Wound healing typically moves through four overlapping stages: hemostasis/coagulation, inflammation, proliferation, and maturation/remodeling [59]. Chronic wounds fail to proceed through a normal, orderly, and timely repair sequence, resulting in delayed wound healing or even non-healing wounds [50]. Chronic wounds are classified by their etiology into four categories: arterial, diabetic, pressure, and venous ulcers [59,60]. Chronic wounds are often companied by high levels of proinflammatory cytokines, persistent infections, the formation of drug-resistant microbial biofilms, and senescent cells that do not respond to repair stimuli [59]. Over the years, chronic wounds have caused great suffering for patients. Non-healing chronic wounds impose physical, psychological, social, and financial burdens on individuals and the broader health system [61]. Reduced angiogenesis is one of the primary causes of the non-healing nature of chronic wounds [50,62]. During the healing process, angiogenesis is an important behavior in the phase of proliferation. Stimulated by moderate hypoxia, cytokines, and protein hydrolases, endothelial cells are activated to proliferate and migrate toward pro-angiogenic signals (such as VEGF and PDGF) to induce angiogenesis [63]. In addition, pericytes and smooth muscle cells can stabilize neovascularization [50]. The new blood vessel network delivers oxygen and nutrients to the damaged tissue and maintains cell function. It can also provide the wound site with cytokines and other substances necessary to repair the damaged tissue. Therefore, promoting angiogenesis and rebuilding tissue blood flow are promising therapeutic targets of new therapies to promote chronic wound healing [50]. Numerous studies have demonstrated that the promotion of angiogenesis can enhance the healing process of chronic wounds [64,65,66,67]. It is worth noting that chronic wounds have a common characteristic: the local deposition of free iron [68]. By chelating iron deposited at the wound site, DFO not only mitigates oxidative stress but also activates the HIF-1α/VEGF pathway (as mentioned previously), thereby facilitating neovascularization and, ultimately, promoting the healing of chronic wounds [69] (Figure 2).

### 2.1. Diabetic Wounds

Persistent hyperglycemia has been shown to detrimentally impact vascular function and elevate susceptibility to infection. Therefore, diabetic wounds frequently do not follow the four stages of wound healing and often develop into chronic wounds [70]. Diabetic foot ulcers are a classic chronic wound [59,60]. Increasing evidence suggests that defective angiogenesis significantly contributes to a delay in diabetic wound healing because damaged blood vessels are unable to deliver critical oxygen and nutrients to the wounded tissue [71]. Thus, promoting angiogenesis is crucial in diabetic wound healing. This process depends on the proliferation and migration of endothelial cells in response to cytokines such as VEGF. As mentioned above, DFO can stimulate the HIF-1α/VEGF pathway to stimulate angiogenesis; therefore, DFO is expected to promote diabetic wound healing.

A recent study investigated the effect of DFO on diabetic wounds. The researchers showed that DFO was able to enhance angiogenesis and accelerate wound healing in diabetic patients by accumulating HIF-1α and regulating endothelial cell function [36]. Dominik Duscher et al. compared the efficacy of the hydroxylase inhibitor dimethyl oxalate (DMOG) and DFO in ameliorating diabetes-related skin wound healing defects by augmenting HIF-1α activation both in vitro and in vivo. The findings demonstrated that DFO effectively stabilized HIF-1α expression in the presence of hypoxia and hyperglycemia, surpassing the impact of DMOG on wound healing and angiogenesis in aged and diabetic mice. These results highlight the significant therapeutic potential of local administration of DFO for diabetic wounds [72].

During the past few years, researchers have worked to use an appropriate approach to enable DFO to perform better in treating diabetic wounds and reducing its side effects. Thus, researchers have taken an interest in utilizing wound dressings loaded with DFO to treat diabetic wounds. Hao Chen et al. utilized DFO-loaded hydrogel nanofibrous scaffolds and a DFO-loaded photo-crosslinked gelatin hydrogel to exploit their potential in promoting diabetic wound healing. The incorporation of DFO into the wound dressing created an optimal microenvironment for cell viability, adhesion, and proliferation. Moreover, the sustained release of DFO significantly enhanced neovascularization. Ultimately, both in vitro and in vivo experiments demonstrated the safety and efficacy of these strategies [73,74]. In addition, the co-delivery of various drugs which have complementary bioactivity provides a better therapeutic strategy for treating diabetic wounds [75]. Due to the synergistic effect of combining DFO and liposome nanoparticles, drug delivery can be enhanced and maintained, thereby amplifying the therapeutic response. Asif Qayoom et al. developed lecithin-based DFO nanoparticles which exhibit superior potential in treating diabetic wounds compared to using DFO alone [76]. Lingzhi Kong et al. demonstrated the synergistic effect of bioglass (BG) (which has been shown to promote vascular regeneration by modulating the expression of VEGF through the inclusion of Si ions) and DFO in promoting revascularization and developed an injectable hydrogel incorporating both BG and DFO for the treatment of chronic diabetic wounds. The findings revealed that the hydrogel exhibited superior efficacy in enhancing wound healing compared to either BG or DFO alone [3]. Bacterial infection and insufficient angiogenesis are the main factors that hinder the healing of diabetic ulcers. Therefore, antimicrobial and angiogenic treatment strategies are key to treating diabetic ulcer wounds. Shan Gao et al. loaded a microneedle patch with the antibacterial drug tetracycline hydrochloride and DFO at the same time, and the prepared microneedle patch not only had good antibacterial properties but also promoted angiogenesis, thus promoting the healing of diabetic ulcer wounds [77]. The in-depth investigation of DFO in diabetic ulcer treatment underscores the pivotal role of angiogenesis in wound repair, thereby providing valuable insights for advancing wound healing therapies [78,79].

### 2.2. Burn Wounds

Burn wounds may secrete a large amount of exudate, increasing excessive inflammation and leading to wound infection, scar formation, and even damage to new blood vessels. Eventually, these wounds may progress into chronic non-healing wounds [80]. The combined action of VEGF, PDGF, and other factors could effectively improve cell (cells involved in skin wound healing and inflammation) function, including proliferation, migration, differentiation, collagen remodeling, etc. Therefore, therapeutic strategies that regulate growth factors at the wound site may promote skin tissue regeneration in burn wounds [81]. Angiogenesis provides nutrients and oxygen to damaged tissues, is essential for maintaining normal cell function, and is an important part of tissue regeneration [47]. Oxidative stress and inflammation may mediate cellular damage and tissue destruction, as the burn wound continues to progress after the abatement of the initial insult [82,83]. Intervening in oxidative stress-induced excitation damage can prevent the progression of partial-thickness second-degree burns to a deep partial-thickness burn or of a deep second-degree burn becoming a third-degree burn [82]. Trace metals such as iron and copper may induce vital cellular injuries via lipid peroxidation [84,85]. Amina El Ayadi et al. treated porcine brass comb burn models with the Livionex formulation (LF) lotion (containing ethylenediaminetetraacetic acid as a metal chelator), and the experimental results showed that the application of LF lotion onto burn wounds provided protection oxidative damage and inflammation and prevented subsequent burn wound progression [82]. Therefore, it is imperative to devise therapeutic strategies that promote angiogenesis and prevent excessive inflammation. DFO can inhibit the activity of PHD, upregulate the expression of HIF-1α, and subsequently stimulate the expression of various growth factors (such as VEGF, PDGF, and SDF-1) [29,30,31,32,33]. In addition, DFO, as an iron-chelating agent, can chelate free iron at the wound site, which is expected to prevent excessive inflammation at the burn wound site and promote wound healing [86,87]. Wu Hongfu et al. developed a hydrogel based on the anti-inflammatory effect of glycyrrhizic acid (GA) and the angiogenic effect of DFO. They demonstrated that the hydrogel effectively reduced pro-inflammatory mediators (TNF-α and IL-6) and upregulated anti-inflammatory mediators (TGF-β3) while promoting proliferation, migration, and angiogenesis of human umbilical vein endothelial cells (HUVECs). Finally, the evaluation of a deep second-degree burn wound model in rats demonstrated that the synthetic hydrogel expedited burn wound healing, providing substantiation for its potential application in treating burn wounds through anti-inflammatory and angiogenesis-promoting mechanisms [80].

### 2.3. Leg Ulcers as the Main Complications of SCD

Leg ulcers are the main complications of sickle cell disease (SCD); about 2.5–40% of SCD patients have the risk of developing leg ulcers because of chronic hemolysis and poor angiogenesis. Leg ulcers are often difficult to heal [88]. As early as 1968, DFO was FDA-approved for chelation of the excess iron produced by hemolysis in SCD patients [89,90]. In order to achieve effective and localized delivery of DFO for ulcer treatment, Melanie Rodrigues developed a novel transdermal delivery system for DFO (DFO-TDDS) that utilizes reverse micelles to ensure continuous delivery of DFO to the skin surface. Rodrigues’ team initially created excision wounds in a transgenic sickle cell mouse model expressing > 99% human sickle hemoglobin (HbSS-BERK); these were subsequently treated with DFO-TDDS. The findings demonstrated that DFO-TDDS significantly expedited wound healing in HbSS-BERK mice by effectively chelating excessive free iron [91]. Their research makes it possible to translate DFO-TDDS into an effective treatment for patients with sickle cell leg ulcers (SCLUs).

## 3. Bone Repair

**Figure 3:**
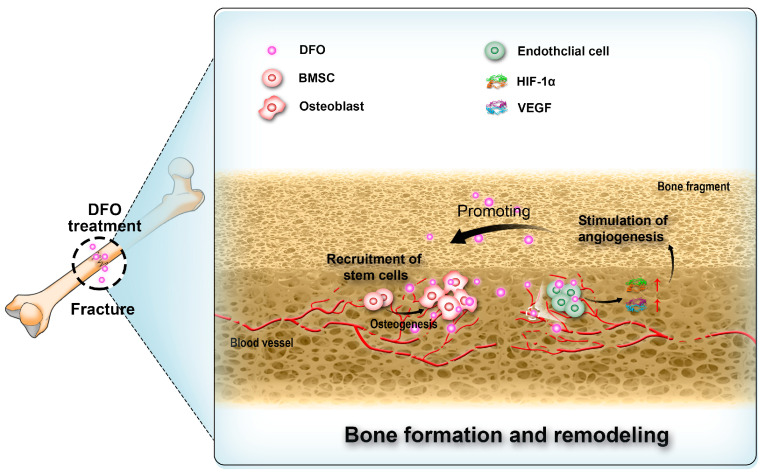
Deferoxamine (DFO) interacts with endothelial cells, bone marrow stem cells (BMSCs), and osteoblasts in the process of bone regeneration.

Angiogenesis is critical for bone regeneration [92,93,94,95,96]. Following fractures, a substantial quantity of locally produced angiogenic growth factors stimulates the process of angiogenesis. These vascular networks not only facilitate the supply of oxygen and nutrients [97,98,99,100,101] but also contribute to the recruitment of bone marrow stem cells (BMSCs) for osteoblastic differentiation and provision of essential ions required for subsequent mineralization stages. Thus, they play a pivotal role in bone regeneration [101,102,103]. Prolyl hydroxylase inhibitors have demonstrated efficacy in activating the HIF-1α pathway [104,105,106], thereby effectively promoting angiogenesis (Figure 3). Thus, DFO as a prolyl hydroxylase inhibitor has been proposed for use in bone repair [107,108,109,110]. Rui Shi et al. co-encapsulated DFO-loaded NPs and free DFO in nanofibers through coaxial electrospinning and investigated its effects on cell viability, migration, and osteogenic differentiation. The results suggested that DFO maintained cell viability and promoted the migration of human mesenchymal stem cells. Alkaline phosphatase (ALP) activity, calcium deposition, and the expression of osteogenesis-related markers and HIF-1α were all increased with DFO, indicating that DFO may accelerate bone regeneration [111].

### 3.1. Distraction Osteogenesis

Distraction osteogenesis (DO) is a technique to initiate regeneration by using mechanical strain to enhance the biological response of injured tissue. It is a metabolic-dependent reconstruction process that relies heavily on adequate local blood supply [112]. However, when distraction osteogenesis is used for bone repair after radiotherapy, the distraction osteogenesis therapy is ineffective due to the reduction of blood vessels [113]. Researchers have confirmed that DFO can optimize the quality and quantity of the regeneration tissue in the sites of mandibular distraction by augmenting vascularity [114,115]. Moreover, in a DO model featuring radiation-induced impairment of bone healing, angiogenesis, and biomechanical properties, DFO has demonstrated the ability to restore vascularity to the distraction site, thereby counteracting the detrimental effects caused by radiation therapy (XRT) and facilitating bone regeneration [108,116,117]. The findings presented here enhance the potential utility of vascular enhancement as a means to optimize bone regeneration in DO.

### 3.2. Steroid-Induced Osteonecrosis of the Femoral Head

Steroids can reduce the expression of VEGF and disrupt vascularization [118]. Therefore, addressing angiogenesis is critical for the treatment of steroid-induced osteonecrosis of the femoral head (ONFH). Jia Li et al. first reported that local DFO administration can improve angiogenesis and bone repair in early-stage models of rabbit ONFH, which may be an efficient, economical, and facile method to treat early-stage ONFH [119].

### 3.3. Bone Defects

In recent years, researchers have made fresh attempts to use DFO treatment in bone repair. Biomimetic materials produced by 3D printing offer a good treatment method for bone transplantation after major defects, and they also make up for the disadvantages of bone autografting [120]. Although the scaffold-based approach has a great therapeutic potential, it relies on the construction of new blood vessels for regeneration; thus, induction of neovascularization at the site of regeneration is crucial [121]. Justin Drager et al. used 3D technology to print biomimetic materials which were transplanted into a rabbit model of bone segmental defect and, through local injection of DFO, increased the formation of blood vessels at the injured site, creating an environment conducive to bone repair [120]. The findings present a novel concept for the design of bone scaffolds with potential for vascularization.

## 4. Lung and Airway

### 4.1. Bronchopulmonary Dysplasia

**Figure 4:**
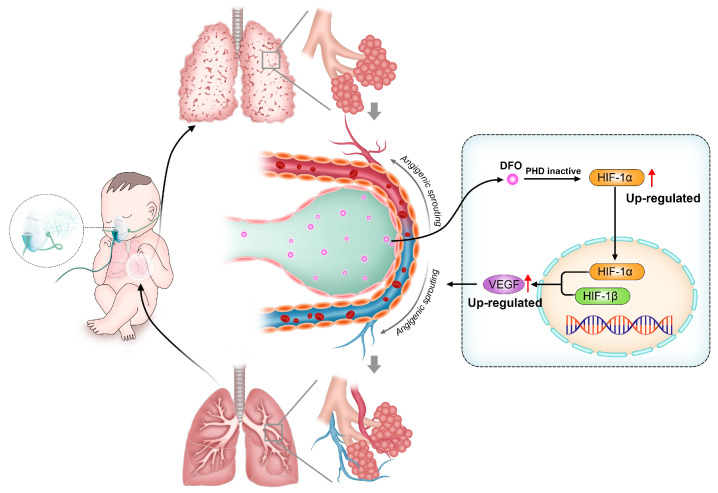
In bronchopulmonary dysplasia (BPD) models, deferoxamine (DFO) promotes angiogenic sprouting by regulating hypoxia-inducible factor-1α (HIF-1α) signaling pathways.

Bronchopulmonary dysplasia (BPD) is a frequent complication in premature infants which seriously affects the health of children. Fetal lungs undergo development in a hypoxic intrauterine environment where HIF-1α plays a crucial role in promoting normal organ growth and maturation. However, premature exposure to oxygen reduces its expression in preterm infants, hindering alveolar and angiogenic processes while disrupting pulmonary development [122,123,124,125]. PHD inhibitors have been shown to promote pulmonary angiogenesis in BPD primate models by increasing HIF-1α and downstream angiogenic factors (Figure 4) [126,127]. As a PHD inhibitor, DFO has been shown to improve lung development in BPD rats by accumulating HIF-1α [128]. Yanru Chen et al. verified, in a mice BPD model, that deferoxamine-loaded aerosol particles (DFO@APs) can release DFO in the alveolar interstitium, thus promoting the reconstruction of microvasculature and, ultimately, inducing lung development for treating BPD [129]. 

### 4.2. Complications of Lung Transplantation

Lung transplantation is often necessary for the treatment of various end-stage lung diseases; however, the occurrence of donor bronchial ischemia poses a significant risk for the development of airway anastomotic complications, potentially leading to severe postoperative complications and transplant failure. Therefore, it is important to promote microvascular repair and alleviate allograft ischemia and hypoxia [130]. Xinguo Jiang et al. developed a DFO nanoparticle and confirmed its ability to improve mouse orthotopic tracheal transplant model complications by producing angiogenic growth factors and reducing ROS production, suggesting that the use of DFO is an effective strategy to reduce postoperative complications following lung and airway transplantation [131]. 

## 5. Spinal Cord Injury

Spinal cord injury (SCI) is a serious traumatic disease. As we know, iron overload, reactive oxygen species accumulation, lipid peroxidation, and glutamate accumulation are all associated with spinal cord injury and are also inducers of ferroptosis (ferroptosis is a regulated form of cell death characterized by iron-dependent phospholipid peroxidation) [132,133,134,135,136]. As an iron death inhibitor, DFO can promote the recovery of spinal cord injury by inhibiting iron death [134,137]. Despite the fact that the therapeutic effect of DFO on SCI has been demonstrated in previous studies, the exact mechanism of action is still controversial [138,139]. Guoqing Tang et al. hypothesized that DFO improves spinal cord compression by promoting angiogenesis and demonstrated, in a moderately compressed SCI rat model, that DFO-induced revascularization via activation of the HIF-1α/VEGF pathway is a key mechanism for improving prognosis in spinal cord injury [140]. In addition, the influx of erythrocytes caused by hemorrhage during SCI provides abundant iron sources at the site of the injury, and the increase in iron concentration, iron metabolism, and superoxide metabolism promote each other, producing a large number of free radicals, mediating the oxidative stress response that contributes to secondary injury [141,142]. Many scholars have studied secondary injury responses, among which the inflammatory cascade caused by tumor necrosis factor-α (TNF-α) is considered to be the core of the secondary injury method [143]. Hence, controlling the inflammatory response is essential for treating SCI and preventing further injury. The potential mechanism of DFO in suppressing the inflammatory response following SCI involves chelation of locally produced iron from bleeding, thereby inhibiting TNF-α and interleukin-1β (IL-1β) production by macrophages and microglia. This subsequently promotes the polarization of macrophages from M1 to M2 phenotype, ultimately leading to inhibition of secondary SCI injury [144,145]. Taken together, these results show that DFO treatment reduces the development of inflammation and tissue injury associated with spinal cord trauma. This may accelerate the clinical application of DFO in SCI.

## 6. Others

During recent years, research on DFO promoting angiogenesis has become increasingly popular. Some researchers use DFO to treat traumatic brain injury. DFO can not only chelate excessive iron from bleeding to prevent oxidative damage through the blood–brain barrier, it can also achieve this through accumulating HIF-1α in order to promote the expression of VEGF, subsequently improving hypoxia tolerance and promoting angiogenesis [146]. DFO has achieved results in the investigation of salivary gland and mammary gland injury reconstruction and in increasing vascularization of islet transplantation due to angiogenesis of DFO [147,148]. In a study of fat transplantation, the experimental results showed that DFO-pretreated adipose fat significantly improved the postoperative weight/volume retention rate, suggesting that DFO promoted angiogenesis in the grafts [149].

## 7. Drug Delivery System

Since DFO is a low-molecular-weight, water-soluble drug with a short retention time in blood vessels, it is necessary to develop a DFO release system to achieve targeted DFO delivery [150]. 

The penetration of DFO through the intact cuticle is essential for achieving the objective of preventing and treating diabetic ulcers [65,150]. Hence, Dominik Duscher et al. encapsulated DFO with nonionic surfactants and polymers to form reverse micelles, which were then dispersed within a release-controlling polymer matrix patch. This enabled the delivery of DFO through the hydrophobic stratum corneum, ensuring its targeted delivery to the dermis. The experimental results demonstrated the efficacy of the transdermal drug delivery system in preventing diabetic pressure ulcers and promoting the healing process of existing diabetic wounds [65]. In 2019, Dominik Duscher et al. used state-of-the-art surface micro-texturing technology to develop an enhanced TDDS (eTDDS). Micro-textured surfaces ensure that the patch contacts the wound bed and increases drug release. The results showed that the improved transdermal delivery system not only released DFO continuously but also had a stronger skin penetration ability. Compared with other delivery methods (drip-on aqueous solution and degradable polymer spray application), DFO eTDDS accelerated healing [150]. With the continuous progress of drug delivery systems, in addition to the enhancement of targeted drug penetration [65,150,151], the combination of DFO and local drug delivery systems—which can not only provide therapeutic payloads but also promote wound healing—has attracted widespread attention [152,153]. Electrospinning is an advanced method used for developing wound dressings [154,155,156,157,158]. A wound dressing made from electrospun fibers can maintain a moist environment, absorb wound secretions, or provide adequate oxygen [67,159,160]. These types of porous scaffoldings have a high surface-area-to-volume ratio, use a hydrophilic polymer, and can load drugs and other bioagents as active components. Mohammad Hossein Kazemi et al. used the electrospinning technique to produce a fiber mat loaded with DFO and ciprofloxacin which was verified in vitro to promote wound healing [161]. 

In addition, the combination of DFO and hydrogels with a porous structure [162,163,164] that can mimic the structure and function of extracellular matrix and promote cell migration, proliferation, and maturation provides a new strategy for the treatment of diabetic ulcers [165,166,167]. For instance, Haijun Shen et al. developed a biomimetic hydrogel containing copper sulfide (CuS) nanoparticles and deferoxamine. DFO and CuS nanoparticles were incorporated into a biomimetic hydrogel which mimics the structure and function of the extracellular matrix. This biomimetic hydrogel can promote cell adhesion and migration, be degraded by cell-secreted matrix metalloproteinases (MMPs), and then release DFO and CuS nanoparticles at the wound site, where they can exert their therapeutic effects. Meanwhile, it can stimulate angiogenesis, effectively eradicate drug-resistant bacteria, and facilitate cell adhesion and migration, all of which are pivotal factors for the healing of diabetic ulcers [78]. An increasing number of studies have shown that changing the administration of DFO can effectively promote angiogenesis and tissue reconstruction [168,169,170]. There is also some evidence that continuous release of DFO or prolongation of the half-life of DFO through the design of a stable drug delivery system can promote cell proliferation and migration and stimulate the formation of blood vessels, providing a theoretical basis for the application of DFO in bone repair [110,171,172,173,174,175,176,177]. Yahong Li et al. used zeolitic imidazolate framework-8 (ZIF-8), which can promote osteogenesis and bone regeneration [178], as a carrier to extend the half-life of DFO. This not only prolonged the drug release but also achieved a synergistic enhancement effect in promoting H-type vessels, angiogenesis, and osteogenic coupling. This provides a new therapeutic strategy, which has a better effect on bone repair, for the regeneration of bone defects of critical size [178]. In the treatment of BPD, DFO is transported into the alveolar interstitium by respiratory delivery, thereby promoting microvascular reconstruction and, ultimately, inducing lung development. In summary, advanced drug delivery systems provide a promising strategy for achieving targeted therapy and improving therapeutic efficacy. This section focuses on exploring the therapeutic effects of diverse delivery systems for DFO in various diseases, aiming to optimize the efficacy of DFO (Table 1).

## 8. Conclusions and Future Perspectives

Deferoxamine can not only be used as an iron chelator to treat iron overload diseases but can also play an indispensable role in the treatment of angiogenesis deficiency diseases. The intrinsic mechanism of deferoxamine in promoting the therapeutic effect of angiogenesis is closely related to the hypoxia-inducible factor-1α signaling pathway. However, deferoxamine is a drug with a short half-life, small molecular weight, and good water solubility, which limits the durable effect of deferoxamine in angiogenesis. Therefore, in treatment to repair skin and tissue (such as diabetic wounds and burn wounds), researchers coated deferoxamine in various hydrogels or patches and applied them to the repair site. The results showed that the targeted release of deferoxamine can promote endothelial cell proliferation, migration, and angiogenesis, thereby promoting wound healing. In bone regeneration treatment, the combination of biomaterials and deferoxamine can prolong drug release while reducing cytotoxicity. This combination also enriches the function of scaffold materials and plays a role in tissue repair and regeneration, immune regulation, optimization of angiogenesis, and promotion of bone tissue regeneration. In addition, an increasing number of studies have shown that deferoxamine has toxic effects in wound healing, such as visual toxicity and osteotoxicity [179,180,181,182]. Therefore, challenges persist regarding how to control the dosage of deferoxamine and improve the mode of administration. Polyelectrolyte capsules have captured our interest in the context of our current research. These polyelectrolyte capsules are believed to be promising drug delivery systems against cancer and are also utilized in self-healing coatings [183]. They are currently undergoing mass production using automated systems at the first stage. These capsules also constitute a promising platform for deferoxamine drug delivery. In addition, the poly (lactic acid) (PLA) microchamber array (MCA) is a biodegradable and biocompatible controlled drug-release system sensitive to the high-intensity focused ultrasound. The synthesis of such arrays has minimal impact on the drug, preserving the drug’s biological properties. This system can open at therapeutic parameters of ultrasound exposure and complete degradation once the drug is released in full [184]. In conclusion, the combination of different drug delivery systems is expected to maximize the potential advantages of deferoxamine in regenerative medicine treatment.

## Figures and Tables

**Table 1:** Different DFO delivery systems.

Delivery System	Composition	Properties	Model Used	Application	Reference
Transdermal drug delivery system	PVP, polymer ethyl cellulose	This approach combines reverse micelle encapsulation of DFO by nonionic surfactants with dispersion in a degradable slow-release matrix, which allows for the targeted delivery of DFO molecules to the dermis.	db/db mice (pressure ulcer model)	Diabetic pressure ulcer	[65]
Injectable hydrogel	SFNs	It can be administered in a locally targeted and minimally invasive manner, and sustained drug release lasts for 40 days.	Diabetic rats (full-thickness wounds)	Diabetic wound healing	[79]
Biomimetic hydrogel	MMP-degradable peptide, HA, RGD	It mimics the structure and function of the extracellular matrix to promote cell adhesion and migration, is degraded by cell-secreted matrix metalloproteinases (MMPs), and subsequently releases the drug at the wound site.	Diabetic mice (full-thickness wounds)	Diabetic wound healing	[78]
Electrospun mat	SF, Ch, PVA	The substance exhibits low toxicity, possesses hemostatic and antimicrobial properties, enables sustained drug release for a duration of 72 h, and facilitates cell adhesion.		Wound healing	[161]
Microneedle patch	HA, Ch, SF	It can destroy biofilms and deliver drugs at a deeper level.	Diabetic rat (full-thickness wounds)	Wound healing	[77]
Electrospun artificial Periosteum	PCL	It can support cell attachment, proliferation, and migration by mimicking the shape and structure of the extracellular matrix. It can be continuously and slowly released for more than 21 days.		Osteogenesis	[111]
Biomimetically hierarchical scaffold	MnCO nanosheets, gelatin- methacryloyl hydrogel, polylactide/HA matrix	With a well-organized gradient structure, it mimics the cortical and cancellous bone tissues; meanwhile, the hydrogels inside the scaffolds provide the scaffolds with additional extracellular matrix characteristics.	Rat femur defect model	Bone regeneration	[171]
Drug-delivery nanoplatform	ZIF-8	The excellent biocompatibility, high porosity, and adjustable pore size of ZIF-8 make it a suitable carrier for encapsulating DFO to extend the half-life of DFO. Moreover, ZIF-8 itself can promote osteogenesis and bone regeneration.	Cranial defect models of rats	Bone regeneration	[178]
Injectable temperature-sensitive hydrogel	GMs, type Ⅰ, collagen, fibronectin	GMs possess long-term release characteristics of DFO, hydrogel that allows the material to automatically adapt to the three-dimensional structure of the defect site, and components similar to the extracellular matrix that promote repair-related cells.	Rat femur critical bone defect model	Bone regeneration	[110]
Aerosol particles	lactic-co-glycolic acid, membranes of macrophages	Its optimized size and the shell–core structure endow aerosol particles with Brownian motion and atomization stability, thus enabling the aerosol particles to reach the bronchi and alveoli deeply for effective deposition.	C57BL/6 mice (oxygen-induced BPD model)	Alveolar reconstruction and lung development	[129]

SF: silk fibroin; Ch: chitosan; PVA: polyvinyl alcohol; SFNs: SF nanofibers; HA: hyaluronic acid; MMP: matrix metalloproteinases; PCL: polycaprolactone; MnCO: manganese carbonyl; ZIF-8: zeolitic imidazolate framework-8; PVP: polymer polyvinylpyrrolidone; GMs: gelatin microspheres.

## Data Availability

No new data were created or analyzed in this study. Data sharing is not applicable to this article.
